# Association Between Dietary Vitamin Intake and Short Sleep Duration Among Adults in the United States: A Cross-Sectional Analysis of 2021-2023 National Health and Nutrition Examination Survey (NHANES) Data

**DOI:** 10.7759/cureus.117247

**Published:** 2026-09-30

**Authors:** Evan N Weber, Hannah J Grimmett, Graham Grunsky, Yuvraj Aggarwal, Chrystal A Sumrall

**Affiliations:** 1 Department of Neuroscience, University of Evansville, Evansville, USA; 2 Department of Anatomical Sciences, William Carey University College of Osteopathic Medicine, Hattiesburg, USA; 3 College of Osteopathic Medicine, William Carey University, Hattiesburg, USA; 4 Family Medicine, Hattiesburg Clinic, Laurel, USA

**Keywords:** dietary vitamin intake, micronutrients, nhanes, short sleep duration, sleep health

## Abstract

Objective

Short sleep duration (SSD) and inadequate dietary vitamin intake are both relevant to overall health and may share relationships with chronic disease risk. However, the relationship between dietary vitamin intake and sleep duration remains incompletely characterized, particularly when vitamin intake is evaluated relative to total energy consumption; additionally, limited research has been done to evaluate the relationship between SSD and vitamins that are not traditionally associated with sleep. Understanding these associations may help clarify potential nutritional factors related to sleep duration. This study evaluated associations between SSD and dietary intake of vitamins A, B1, B2, B3, B6, B9, B12, C, D, E, and K per 1,000 kilocalories among adults in the United States.

Materials and methods

This cross-sectional study used 2021-2023 National Health and Nutrition Examination Survey (NHANES) data. Adults aged ≥20 years with complete sleep, dietary, covariate, and Day 1 dietary weight data were included (n=4,169). SSD was defined as fewer than 7 hours of usual weekday/workday sleep. Primary endpoints were vitamins A, B6, and K; all other vitamins were secondary endpoints. Participants were categorized into low, middle, and high intake tertiles for each vitamin. Separate weighted logistic regression models were performed using the NHANES Day 1 dietary sample weight and adjusted for age, sex, race/ethnicity, education, income-to-poverty ratio, total energy intake, and body mass index. NHANES strata and primary sampling units were not incorporated into the analysis.

Results

SSD prevalence decreased across low-to-high intake groups for vitamin A (24.2% to 16.4%), vitamin B6 (22.7% to 18.7%), and vitamin K (23.5% to 17.8%). Higher intake remained associated with lower odds of SSD for vitamin A (aOR=0.710, 95% CI 0.710-0.711), vitamin B6 (aOR=0.724, 95% CI 0.723-0.725), and vitamin K (aOR=0.815, 95% CI 0.814-0.816). Weaker inverse associations were observed for vitamins B1, B2, B3, B12, D, and E. Vitamin C demonstrated a nonlinear inverse pattern, whereas vitamin B9 showed a direct association.

Conclusion

Higher dietary intake of vitamins A, B6, and K was associated with lower odds of SSD among adults in the United States. Causation cannot be inferred because of the cross-sectional study design. In addition, confidence intervals and significance estimates should be interpreted cautiously because NHANES strata and primary sampling units were not incorporated into the analysis. Prospective studies using full complex survey methods are warranted to further evaluate these associations.

## Introduction

Sleep-related complaints are commonly reported among adults in the United States and may include insomnia, daytime fatigue, and other disturbances that affect the quality or duration of sleep [1]. Short sleep duration (SSD) is defined as fewer than seven hours of sleep within a 24-hour period and is particularly important because most health organizations recommend at least seven hours of sleep per night to maintain optimal health in adults [2-4]. Within the United States, SSD affects approximately 33.2% of adults, highlighting its relevance as a public health concern [3].

SSD has been associated with several adverse health outcomes. From a metabolic perspective, insufficient sleep has been linked to impaired glucose tolerance, insulin resistance, increased ghrelin, and decreased leptin; each of which may contribute to altered appetite regulation and increased cardiometabolic risk [1,5]. Cardiovascularly, SSD has been associated with a greater risk of hypertension, stroke, coronary artery calcification, and coronary heart disease; this is thought to occur via inflammatory pathways and elevated markers such as C-reactive protein [1,6-8]. Psychologically, SSD has also been correlated with anxiety, depression, and hypomania [1,9]. Stress can disrupt sleep by increasing cognitive and physiological arousal, whereas insufficient or disturbed sleep may intensify neuroendocrine stress responses, creating a bidirectional cycle. Chronic stress may also increase adrenal cortisol production and reduce vitamin D receptor expression, potentially impairing vitamin D uptake and activation; this suggests a possible association among psychological stress, sleep disturbance, and vitamin D deficiency [6]. Given these broad health implications, identifying modifiable factors associated with SSD may help guide clinical strategies aimed at improving sleep health and reducing chronic disease risk.

Despite the frequency of sleep complaints, conservative management and lifestyle modifications may be underutilized, forcing many patients to rely on pharmacologic or supplemental sleep aids. In 2024, a broad measure of sleep-aid use found that 12.9% of U.S. adults reported using prescription medications, over-the-counter medications or supplements, or tetrahydrocannabinol products for sleep [10]. Sleep disorders have also been estimated to account for approximately $94.9 billion in incremental healthcare costs in the United States [11]. Therefore, lifestyle-based interventions, including patient education on sleep hygiene, diet, alcohol and tobacco use, and regular physical activity, represent important facets of sleep health management that may result in lower overall sleep-related healthcare costs [12]. 

Diet is another modifiable lifestyle factor with substantial implications for chronic disease prevention. Vitamins are essential micronutrients involved in numerous metabolic, neurologic, immune, hematologic, and cardiovascular processes [13,14]. Because most vitamins cannot be synthesized in adequate amounts by the body, sufficient dietary intake is necessary to support normal physiologic function [15]. Inadequate vitamin intake, even when not severe enough to cause classic deficiency symptoms, may contribute to poorer overall health and increased chronic disease risk [15,16].

Although vitamins A, B6, and K have not been widely studied in relation to sleep, they have unique and diverse roles in physiologic pathways that may influence both sleep and cardiometabolic health [17,18]. Vitamins A and K are both fat-soluble vitamins; vitamin A is involved in vision, cellular differentiation, and immune function, while vitamin K is most recognized for its role in the coagulation cascade, but has also been studied in relation to broader metabolic and inflammatory processes [17]. Vitamin B6 is a water-soluble vitamin that serves as a cofactor in numerous enzymatic reactions, including pathways related to neurotransmitter metabolism [18]. Other vitamins, including B1, B2, B3, B9, B12, C, D, and E, also contribute to essential physiologic functions and may provide additional insight into the relationship between dietary micronutrient intake and sleep duration [17-19].

Although poor diet and poor sleep have each been associated with adverse health outcomes, the relationship between dietary vitamin intake and sleep duration remains insufficiently characterized. This study aims to address this gap by using 2021-2023 National Health and Nutrition Examination Survey (NHANES) data to evaluate the association between SSD and dietary intake of selected vitamins among U.S. adults, with primary emphasis on vitamins A, B6, and K and secondary evaluation of vitamins B1, B2, B3, B9, B12, C, D, and E; vitamins A, B6, and K were prioritzed over the other, aforementioned vitamins, as there is very little research discussing their involvement in sleep patterns. Assessing this relationship may provide physicians with a broader evidence base for counseling patients with SSD on lifestyle modification and dietary habits; this supports both early intervention in sleep health management and potentially reduces some of the healthcare burden associated with sleep-related conditions in the United States.

## Materials and methods

Study design and data source

This cross-sectional study used publicly available data from the 2021-2023 NHANES to evaluate the association between dietary vitamin intake and SSD among adults in the United States. NHANES is a nationally representative survey that collects demographic, dietary, examination, laboratory, and questionnaire data from the noninstitutionalized U.S. population. For this analysis, demographic, dietary recall, sleep questionnaire, and body measures files were merged using the respondent sequence number (SEQN), a unique participant identifier. Because this study used de-identified, publicly available data, institutional review board approval and informed consent were not required for this secondary analysis. This study is reported in accordance with the Strengthening the Reporting of Observational Studies in Epidemiology (STROBE) guidelines for cross-sectional studies [20].

Study population

The analytic sample was restricted to adults aged 20 years or older with complete data for sleep duration, dietary vitamin intake, covariates, body mass index, and Day 1 dietary sample weights. Participants with missing data for any included variables were excluded. Pregnancy and lactation status were not prespecified exclusion criteria in this analysis. Therefore, pregnant or breastfeeding adults who otherwise met the study eligibility criteria may have been included in the analytic sample. Adults older than 60 years were retained in the analytic sample because the study was designed to evaluate the association between dietary vitamin intake and short sleep duration across the adult population rather than within a restricted age range. Because sleep duration and dietary patterns may vary with age, age was included as a covariate in all adjusted regression models. The final adjusted analytic sample included 4,169 adults (Table 1). A formal sample-size calculation was not performed because this was a secondary analysis of an existing NHANES dataset. The analytic sample included all adults aged 20 years or older who met the eligibility criteria and had complete data for the variables included in the analysis.

**Table 1:** Characteristics of the analytic sample (n = 4,169). Continuous variables are presented as weighted median (interquartile range), and categorical variables are presented as unweighted n (weighted %). Weighted estimates were calculated using the  National Health and Nutrition Examination Survey (NHANES) Day 1 dietary sample weight (WTDRD1). NHANES strata and primary sampling unit variables were not incorporated. BMI = body mass index; SSD = short sleep duration; GED: general educational development.

Characteristic	Value
Age, years (IQR)	49 (34–63)
Income-to-poverty ratio (IQR)	3.14 (1.64–5.00)
BMI, kg/m² (IQR)	28.5 (24.8–33.6)
Total energy intake, kcal/day (IQR)	1,914 (1,409–2,502)
Sex, n (weighted %)	
Male	1,835 (48.7%)
Female	2,334 (51.3%)
Race/ethnicity, n (weighted %)	
Mexican American	261 (7.0%)
Other Hispanic	392 (9.2%)
Non-Hispanic White	2,623 (62.8%)
Non-Hispanic Black	457 (10.6%)
Non-Hispanic Asian	179 (5.1%)
Other race, including multiracial	257 (5.3%)
Education, n (weighted %)	
Less than 9th grade	145 (2.4%)
9th-11th grade	287 (5.7%)
High school graduate/GED or equivalent	814 (23.7%)
Some college or associate degree	1,278 (30.2%)
College graduate or above	1,645 (38.0%)
Sleep-duration status, n (weighted %)	
Non-SSD (≥7 hours)	3,325 (79.4%)
SSD (<7 hours)	844 (20.6%)

Study variables

The primary endpoints of interest were dietary intake of vitamins A/retinol, B6/pyridoxine, and K/phytonadione in relation to SSD. Secondary endpoints included dietary intake of vitamins B1/thiamine, B2/riboflavin, B3/niacin, B9/folate, B12/cobalamin, C/ascorbic acid, D/cholecalciferol, and E/tocopherol in relation to SSD. 

SSD was defined as fewer than seven hours of sleep per day. Sleep duration was assessed using the NHANES-derived variable SLD012, which represents the usual number of hours slept on weekdays or workdays and is calculated from self-reported usual weekday/workday sleep time (SLQ300) and wake time (SLQ310). Participants reporting fewer than seven hours of sleep were classified as having SSD, while those reporting 7 or more hours were classified as non-SSD. The official NHANES 2021-2023 Sleep Disorders questionnaire was used to obtain these sleep and wake-time data (see Appendix A). The sleep assessment did not use a specified retrospective recall period, such as the preceding week or month. Rather, participants reported the times they usually fell asleep and woke up on weekdays or workdays, from which NHANES derived usual weekday/workday sleep duration. The present study specifically evaluated sleep duration rather than overall sleep quality; therefore, measures of sleep quality were not incorporated into the SSD definition.

Dietary vitamin intake

Dietary vitamin intake was assessed using the NHANES Day 1 Total Nutrient Intakes file (DR1TOT_L), which reflects nutrient intake from foods and beverages reported during the 24-hour dietary recall covering the previous midnight-to-midnight period. Nutrient intake from dietary supplements, antacids, or medications was not included in the exposure variables. Vitamins included in the analysis were vitamins A, B1, B2, B3, B6, B9, B12, C, D, E, and K. The official NHANES 2021-2023 Dietary Recall questionnaire was used to collect these dietary data (see Appendix B).

To account for differences in total energy intake, each vitamin intake variable was standardized per 1,000 kcal. For each vitamin, participants were categorized into low, middle, and high intake tertiles, with the low intake group used as the reference category in regression analyses (Table 2).

**Table 2:** Dietary vitamin intake tertile cutoffs. Dietary vitamin intake was standardized per 1,000 kcal and categorized into low, middle, and high intake tertiles. Values shown represent the cutoff ranges used to define each intake group. The analytic sample included 4,169 participants. g = grams, mcg= micrograms, mg NE = milligrams of niacin equivalents, mcg DFE = micrograms of dietary folate equivalents, mcg RAE = micrograms of retinol activity equivalents.

Vitamin	Unit	Low intake tertile	Middle intake tertile	High intake tertile
Vitamin A / Retinol	mcg RAE/1000 kcal	≤184.5500	>184.5500 to ≤328.5000	>328.5000
Vitamin B1 / Thiamine	mg/1000 kcal	≤0.6078	>0.6078 to ≤0.8023	>0.8023
Vitamin B2 / Riboflavin	mg/1000 kcal	≤0.7705	>0.7705 to ≤1.0316	>1.0316
Vitamin B3 / Niacin	mg NE/1000 kcal	≤9.0993	>9.0993 to ≤12.5350	>12.5350
Vitamin B6 / Pyridoxine	mg/1000 kcal	≤0.6745	>0.6745 to ≤0.9761	>0.9761
Vitamin B9 / Folate	mcg DFE/1000 kcal	≤166.4300	>166.4300 to ≤240.6400	>240.6400
Vitamin B12 / Cobalamin	mcg/1000 kcal	≤1.2516	>1.2516 to ≤2.2185	>2.2185
Vitamin C / Ascorbic Acid	mg/1000 kcal	≤16.3700	>16.3700 to ≤47.6300	>47.6300
Vitamin D / Cholecalciferol	mcg/1000 kcal	≤1.0124	>1.0124 to ≤2.5000	>2.5000
Vitamin E / Tocopherols	mg alpha-tocopherol/1000 kcal	≤3.3077	>3.3077 to ≤5.0947	>5.0947
Vitamin K / Phytonadione	mcg/1000 kcal	≤28.5600	>28.5600 to ≤57.5000	>57.5000

Covariates

Models were adjusted for age, sex, race/ethnicity, education level, income-to-poverty ratio, total energy intake, and body mass index (BMI). These covariates were selected to account for demographic, socioeconomic, dietary, and anthropometric factors that may be associated with both dietary intake and sleep duration. Specific medical comorbidities were not included as covariates in the present models. The analysis focused on prespecified demographic, socioeconomic, dietary, and anthropometric covariates; therefore, potential confounding by chronic medical conditions was addressed as a study limitation rather than through additional disease-specific adjustment. Occupation was not included as a covariate in the present analysis. The adjusted models were based on prespecified demographic, socioeconomic, dietary, and anthropometric factors. Because occupational characteristics, including work schedule and shift work, may influence sleep duration, the absence of occupational adjustment represents a potential source of residual confounding.

Statistical analyses

Weighted descriptive statistics were used to characterize the analytic sample. Crosstabulations and chi-square tests were performed to evaluate differences in SSD prevalence across low, middle, and high intake groups for each vitamin. Fully adjusted weighted logistic regression models were then used to examine the association between vitamin intake groups and SSD.

Participants with missing data for any variable included in the adjusted models were excluded using a complete-case approach. No imputation of missing data was performed. No sensitivity or interaction analyses were performed. No formal adjustment for multiple comparisons was applied; therefore, secondary analyses were considered exploratory.

Primary analyses focused on vitamins A, B6, and K. Secondary exploratory analyses examined vitamins B1, B2, B3, B9, B12, C, D, and E. Each vitamin was modeled separately using fully adjusted weighted logistic regression. Adjusted odds ratios (aORs) and 95% confidence intervals (CIs) were calculated for the middle and high intake groups compared with the low intake group.

Analyses were conducted using IBM SPSS Statistics for Windows, version 30 (IBM Corp., Armonk, N.Y., USA). The Day 1 dietary sample weight variable (WTDRD1) was applied to account for dietary recall weighting. However, because NHANES stratification and clustering variables were not incorporated, confidence intervals and p-values were interpreted cautiously.

## Results

The merged 2021-2023 NHANES dataset included 11,933 participants, of whom 7,809 were aged 20 years or older. Among these adults, 7,698 had complete weekday/workday sleep-duration data, and 4,754 also had complete dietary vitamin-intake data. After excluding 585 participants with missing family income-to-poverty ratio and/or body mass index data, 4,169 participants remained in the final analytic sample. Because a complete-case approach was used, there were no missing values for variables included in the analyses among the final 4,169 participants.

Dietary vitamin intake was standardized per 1,000 kcal and categorized into low, middle, and high intake groups for each vitamin (Table 2).

SSD by vitamin intake group

For the primary vitamin exposures of interest, the weighted prevalence of SSD generally decreased across increasing intake groups. For vitamin A, SSD prevalence was highest in the low intake group at 24.2%, followed by 20.5% in the middle intake group and 16.4% in the high intake group. A similar pattern was observed for vitamin B6, with SSD prevalence decreasing from 22.7% in the low intake group to 20.1% in the middle intake group and 18.7% in the high intake group. For vitamin K, SSD prevalence decreased from 23.5% in the low intake group to 20.2% in the middle intake group and 17.8% in the high intake group (Figure 1).

**Figure 1:**
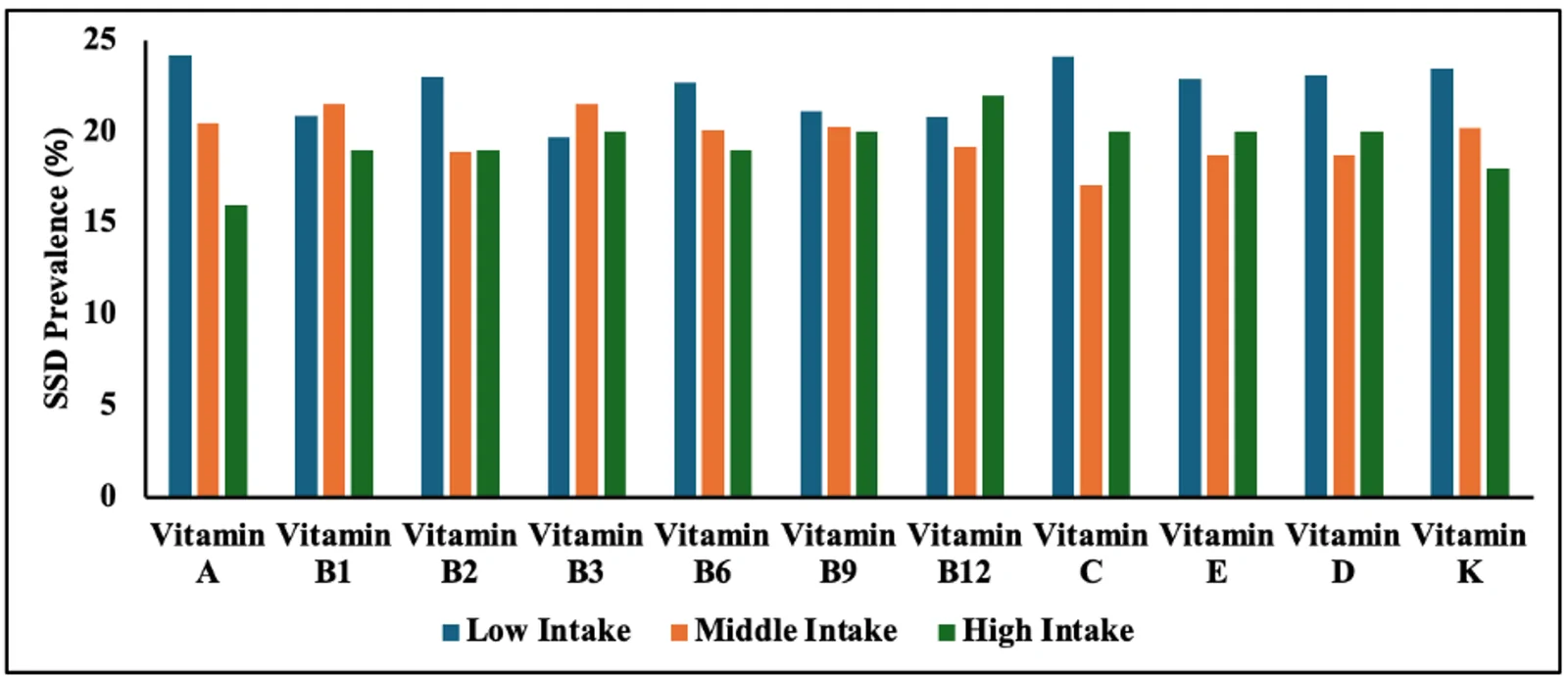
Weighted prevalence of SSD by vitamin intake tertile. Figure created using Microsoft Excel (Microsoft Corporation, Redmond, WA, USA). SSD = short sleep duration.

Among the secondary vitamin endpoints, several vitamins demonstrated lower SSD prevalence in the middle or high intake groups compared with the low intake group. Vitamin C showed the lowest SSD prevalence in the middle intake group, decreasing from 24.1% in the low intake group to 17.1% in the middle intake group, with a prevalence of 20.0% in the high intake group. Similar patterns were observed for vitamins D and E, where SSD prevalence was 23.1% and 22.9% in the low intake groups, respectively, compared with 18.7% in the middle intake groups and 20.0% in the high intake groups. Vitamins B2 and B12 also showed lower SSD prevalence in the middle intake groups compared with the low intake groups, although the high intake groups did not demonstrate a consistent monotonic decrease. Vitamins B1, B3, and B9 demonstrated smaller differences in SSD prevalence across intake groups (Figure 1).

Adjusted association between vitamin intake and SSD

In fully adjusted weighted logistic regression models, higher intake of the primary vitamins A, B6, and K was associated with lower odds of SSD compared with the low intake reference group. For vitamin A, the middle intake group had lower adjusted odds of SSD compared with the low intake group, although the magnitude of association was modest (aOR, 0.909; p<0.001). The high intake group demonstrated a stronger inverse association with SSD (aOR, 0.710; p<0.001). For vitamin B6, both the middle and high intake groups were associated with substantially lower odds of SSD compared with the low intake group, with adjusted odds ratios of 0.780 and 0.724, respectively (both p<0.001). For vitamin K, the middle intake group had modestly lower odds of SSD (aOR, 0.914; p<0.001), while the high intake group had a stronger inverse association (aOR, 0.815; p<0.001) (Table 3).

**Table 3:** Adjusted odds ratios for SSD by vitamin intake tertile. Values are presented as adjusted odds ratios (aORs) with 95% confidence intervals. The low-intake tertile served as the reference group. Models were adjusted for age, sex, race/ethnicity, education, income-to-poverty ratio, total energy intake, and body mass index. The National Health and Nutrition Examination Survey (NHANES) Day 1 dietary sample weight (WTDRD1) was applied; however, strata and primary sampling unit variables were not incorporated. Therefore, confidence intervals and p-values may not fully reflect the complex survey design and should be interpreted cautiously. SSD = short sleep duration.

Vitamin	Low intake group	Middle vs. low aOR (95% CI)	P-value	High vs. low aOR (95% CI)	P-value
						Vitamin A	Reference	0.909 (0.909-0.910)	<0.001	0.710 (0.710-0.711)	<0.001
Vitamin B1	Reference	1.136 (1.135-1.137)	<0.001	0.919 (0.918-0.920)	<0.001						
						Vitamin B2	Reference	0.910 (0.909-0.911)	<0.001	0.877 (0.876-0.878)	<0.001
Vitamin B3	Reference	1.037 (1.036-1.037)	<0.001	0.940 (0.939-0.940)	<0.001						
						Vitamin B6	Reference	0.780 (0.780-0.781)	<0.001	0.724 (0.723-0.725)	<0.001
Vitamin B9	Reference	1.066 (1.065-1.067)	<0.001	1.050 (1.049-1.051)	<0.001						
						Vitamin B12	Reference	0.890 (0.889-0.891)	<0.001	0.955 (0.954-0.956)	<0.001
Vitamin C	Reference	0.627 (0.627-0.628)	<0.001	0.778 (0.778-0.779)	<0.001						
						Vitamin D	Reference	0.819 (0.818-0.819)	<0.001	0.887 (0.886-0.888)	<0.001
Vitamin E	Reference	0.816 (0.815-0.816)	<0.001	0.917 (0.916-0.917)	<0.001						
						Vitamin K	Reference	0.914 (0.913-0.915)	<0.001	0.815 (0.814-0.816)	<0.001

Secondary exploratory analyses demonstrated additional associations between dietary vitamin intake and SSD. Higher intake of vitamins B2, C, D, and E was associated with lower adjusted odds of SSD in both the middle and high intake groups compared with the low intake group. The strongest inverse association among the secondary endpoints was observed for vitamin C in the middle intake group (aOR, 0.627; p<0.001), although the high intake group also had lower odds of SSD compared with the low intake group (aOR, 0.778; p<0.001). Vitamins D and E demonstrated similar patterns, with lower adjusted odds of SSD in the middle intake groups and more modest inverse associations in the high intake groups. Vitamin B2 was also inversely associated with SSD in both the middle and high intake groups, with adjusted odds ratios of 0.910 and 0.877, respectively (both p<0.001) (Table 3).

Associations for vitamins B1, B3, B9, and B12 were less consistent. Vitamin B1 showed higher adjusted odds of SSD in the middle intake group (aOR, 1.136; p<0.001), but lower odds in the high intake group (aOR, 0.919; p<0.001). Vitamin B3 demonstrated a small positive association in the middle intake group (aOR, 1.037; p<0.001) and a modest inverse association in the high intake group (aOR, 0.940; p<0.001). Vitamin B9 was associated with slightly higher adjusted odds of SSD in both the middle and high intake groups compared with the low intake group. Vitamin B12 showed lower adjusted odds of SSD in the middle intake group (aOR, 0.890; p<0.001), but only a modest inverse association in the high intake group (aOR, 0.955; p<0.001) (Table 3).

Overall, higher intake of vitamins A, B6, and K was associated with lower prevalence and lower adjusted odds of SSD among U.S. adults. These findings support a potential relationship between dietary vitamin intake and sleep duration, although results should be interpreted cautiously given the cross-sectional study design and the use of dietary recall data.

## Discussion

In this study, higher dietary intake of vitamins A, B6, and K was associated with lower prevalence and lower adjusted odds of SSD. These associations remained after adjustment for sex, age, race/ethnicity, total caloric intake, BMI, income-to-poverty ratio, and education level; however, residual confounding from measured and unmeasured factors cannot be excluded. Vitamin K demonstrated a somewhat weaker inverse association than vitamins A and B6. Secondary analyses also identified inverse associations for vitamins B2, C, D, and E, although the magnitude and pattern of these associations varied across intake groups. Because of the cross-sectional design, these findings should be interpreted as associations rather than evidence that greater vitamin intake directly reduces the likelihood of SSD. To place the observed intake categories in a broader nutritional context, the Food and Drug Administration (FDA) daily intake recommendations were compared with the vitamin intake categories used in this study (Table 4).

**Table 4:** FDA daily values normalized per 1,000 kcal assuming a 2,000-kcal diet. g = grams, mcg = micrograms, mg NE = milligrams of niacin equivalents, mcg DFE = micrograms of dietary folate equivalents, mcg RAE = micrograms of retinol activity equivalents; FDA: Food and Drug Administration.

Vitamin	Unit	Daily value	Intake per 1,000 kcal
A / Retinol	mcg RAE	900	450
B1 / Thiamine	mg	1.2	0.6
B2 / Riboflavin	mg	1.3	0.65
B3 / Niacin	mg NE	16	8
B6 / Pyridoxine	mg	1.7	0.85
B9 / Folate	mcg DFE	400	200
B12 / Cobalamin	mcg	2.4	1.2
C / Ascorbic Acid	mg	90	45
D / Cholecalciferol	mcg	20	10
E / Tocopherol	mg alpha-tocopherol	15	7.5
K / Phytonadione	mcg	120	60

Of note, NHANES dictated the low, middle, and high intake groups cutoffs that represent dietary vitamin intake rather than utilizing typical measures of vitamin deficiency, adequacy, or excess. For vitamins A and K, the FDA-recommended intake per 1,000 kcal was higher than the cutoff used to define the high intake group; this indicates that individuals who were classified in the high intake group via NHANES may not necessarily have met FDA-recommended intake levels. For vitamin B6, the FDA recommendation fell within the middle intake group; the high intake group was, as a result, consuming more than the recommended amount. These comparisons suggest that individuals categorized as having higher vitamin A and K intake may still fall below FDA-recommended intake levels, whereas individuals in the high vitamin B6 intake group may exceed the FDA recommendation. Clinically, this distinction is important, as it frames the observed associations in relation to practical dietary intake goals rather than deficiency or excess. 

Vitamin A may be particularly relevant to sleep duration because of its established role in visual function. As a fat-soluble vitamin, vitamin A includes retinoid compounds such as retinol, retinal, and retinoic acid; retinal is required for normal photoreceptor function and light perception [17]. Light exposure is also a major environmental signal involved in circadian rhythm regulation and sleep-wake timing [21]. Together, these physiologic roles provide a biologically plausible context for the observed association between higher dietary vitamin A intake and lower odds of SSD; however, the present cross-sectional findings cannot establish a direct mechanistic or causal relationship. Vitamin A also contributes to cellular differentiation, epithelial maintenance, immune function, and gene expression through retinoic acid signaling, suggesting that any relationship with sleep may involve broader physiologic pathways beyond vision alone [17]. Higher vitamin A intake may additionally reflect overall dietary quality or correlated health behaviors rather than an independent vitamin-specific effect. This may be particularly relevant among individuals with reduced fat absorption, restrictive dietary patterns, pancreatitis, alcohol use disorder, or circadian disruption from shift work, all of which may influence vitamin A intake, absorption, or sleep-related physiology [17]. These findings should not be interpreted as support for unsupervised vitamin A supplementation. Vitamin A is fat-soluble and can accumulate in the liver. Although the liver does not have a universally accepted storage threshold for toxicity, concentrations above the adequate range of 0.1 to less than 0.7 µmol/g may be considered elevated, with concentrations of at least 1 µmol/g proposed as an indicator of hypervitaminosis A [22]. Adults are also advised not to chronically exceed the tolerable upper intake level of 3,000 µg retinol activity equivalents per day of preformed vitamin A because excessive intake may increase the risk of hepatotoxicity and skeletal abnormalities [15,17,23]. Accordingly, the present findings support further investigation of dietary vitamin A and sleep duration rather than supplementation as a treatment for SSD. Pregnant individuals should avoid high-dose supplementation with preformed vitamin A because excessive intake is teratogenic; supplementation decisions should be individualized based on vitamin A form and total dietary intake [17].

Vitamin B6 demonstrated one of the strongest inverse associations with SSD among the primary vitamins examined, and its involvement in amino acid metabolism and neurotransmitter-related pathways provides a biologically plausible context for this finding. Vitamin B6 refers to a group of related compounds, including pyridoxine, pyridoxal, and pyridoxamine; pyridoxal 5'-phosphate serves as the biologically active coenzyme form, which is involved in numerous enzymatic reactions [18]. Vitamin B6 is required for the metabolism of tryptophan to serotonin, with a percentage of serotonin being further metabolized to melatonin; both of the aforementioned endogenous neurotransmitters are heavily involved in sleep-wake regulation [18,24]. The biological relevance of this pathway is further supported by the clinical use of melatonin receptor agonists, such as ramelteon, for insomnia [25]. As a result, an increased vitamin B6 intake may reflect greater dietary support for neurochemical pathways that contribute to sleep regulation. These findings may be especially relevant for patients receiving medications that interfere with vitamin B6 metabolism, such as isoniazid, or for individuals who get insufficient levels of vitamin B6 through diet alone [26]. Additional populations at risk for vitamin B6 deficiencies include individuals with impaired renal function or receiving dialysis, people with malabsorptive or autoimmune disorders, and those with alcohol dependence. These findings should not be interpreted as support for unsupervised high-dose vitamin B6 supplementation, as chronically high pyridoxine intake over a prolonged period of time has been associated with other side effects, most commonly sensory peripheral neuropathy [26].

While vitamin K is not typically considered when discussing sleep physiology, it also demonstrated an inverse association with SSD. As a fat-soluble vitamin, vitamin K is best recognized for its role in coagulation, as it is required for the activation of vitamin K-dependent clotting factors [17]. Beyond coagulation, vitamin K-dependent proteins are also involved in physiologic processes related to bone metabolism, vascular calcification, and broader cardiometabolic health; this suggests that vitamin K may overlap with pathways relevant to chronic disease risk [27]. For example, vitamin K has been implicated in cardiovascular health, while SSD has also been associated with cardiometabolic outcomes, including hypertension, stroke, coronary heart disease, obesity, and diabetes [7,8,27]. Therefore, the observed association between vitamin K intake and SSD may reflect broader relationships between sleep duration, diet quality, and cardiometabolic health rather than a direct sleep-specific mechanism. Vitamin K intake may also serve as a marker of dietary quality because its derivatives, particularly phylloquinone, are commonly obtained from leafy green vegetables and other nutrient-dense plant foods [28]. As a result, these findings may be especially relevant for patients with low intake of leafy green vegetables, fat malabsorption, or cardiometabolic risk factors, as vitamin K intake or metabolism may be decreased or abnormal, respectively. Patients who have undergone bariatric surgery or who receive prolonged antibiotic therapy may also warrant consideration because vitamin K absorption or intestinal availability may be reduced [29,30]. Patients taking warfarin represent another clinically important population. Rather than eliminating vitamin K-rich foods, these patients generally require a consistent vitamin K intake because abrupt changes in dietary or supplemental intake can alter anticoagulant effects [31]. 

Secondary exploratory analyses demonstrated that the relationship between dietary vitamin intake and SSD was not uniform across all vitamins examined (Table 3). Unlike vitamins A and K, the FDA recommendations for vitamins B2 and B3 fell below the lower cutoff for the low intake group, indicating that most participants meeting the recommended intake would already exceed the lowest intake category. Conversely, the FDA recommendations for vitamins C and B9 fell within the middle intake group, while vitamins D and E exceeded the cutoff for the higher intake group. These findings suggest that the relationship between dietary recommendations and the observed SSD risk differs across vitamins and that FDA-recommended intake may correspond to different exposure categories depending on the nutrient or vitamin examined. 

The observed associations may also reflect factors beyond the individual vitamins examined. Dietary vitamin intake is closely related to food choice, nutrient density, and overall dietary patterns; therefore, higher intake of a particular vitamin may function partly as a marker of broader diet quality or correlated health behaviors rather than an independent vitamin-specific effect. Reverse causation is also possible, as individuals with shorter or poorer sleep may have different dietary patterns, meal timing, or food choices that influence vitamin intake. In addition, residual confounding from factors not incorporated into the adjusted models, including physical activity, smoking, alcohol and caffeine intake, supplement use, chronic disease, medication use, occupation, and shift work, cannot be excluded. Accordingly, the observed associations should not be interpreted as evidence that increasing intake of any individual vitamin would improve sleep duration.

The potential clinical relevance of these findings should be considered cautiously. Patients presenting with SSD may have overlapping lifestyle, metabolic, psychological, dietary, and medication-related factors that contribute to sleep health [1]. Current management of sleep complaints commonly includes evaluation for underlying causes, sleep hygiene counseling, behavioral modification, and, when appropriate, pharmacologic therapy [10,12,32]. The associations observed in this study suggest that dietary patterns may warrant further investigation as one component of the broader relationship between lifestyle and sleep duration. However, these findings do not establish that modifying vitamin intake will improve sleep duration or reduce sleep-related health risks. Future prospective and interventional studies are needed before vitamin-specific dietary recommendations can be made for the management of SSD.

With respect to limitations, the cross-sectional design prevents conclusions regarding causality or directionality, and reverse causation cannot be excluded. Sleep duration was self-reported as usual weekday/workday sleep and may not capture sleep quality, weekend patterns, insomnia symptoms, obstructive sleep apnea, circadian disruption, or objectively measured sleep. Dietary vitamin intake was based on a single 24-hour recall and may not reflect usual long-term intake, circulating vitamin concentrations, or clinical deficiency status. Because vitamins were modeled separately, observed associations may reflect correlated food sources, overall diet quality, or related health behaviors rather than independent vitamin-specific effects. Residual confounding also remains possible because occupation and shift work, physical activity, smoking, caffeine and alcohol intake, medication use, chronic disease, psychiatric symptoms, food insecurity, and supplement use were not fully incorporated into the models. Pregnancy and lactation were not exclusion criteria or covariates and may influence both sleep and dietary patterns. Although age was included as a covariate, residual age-related differences may remain. Day 1 dietary sample weights were applied; however, NHANES strata and primary sampling unit variables were not incorporated, so variance estimates, confidence intervals, and p-values should be interpreted cautiously. Given the number of vitamin exposures and comparisons examined, particularly among secondary endpoints, chance findings related to multiple testing are also possible. Finally, because NHANES samples the noninstitutionalized U.S. population, these findings may not generalize to institutionalized populations or populations outside the United States.

Future research should further evaluate the relationship between dietary vitamin intake and sleep duration using prospective and interventional study designs. Studies incorporating repeated dietary recalls, serum or plasma vitamin biomarkers, objective sleep measures such as actigraphy, and full complex survey methods would help clarify whether the observed associations reflect true vitamin-specific relationships, broader dietary patterns, or residual confounding. Additional research should also examine whether these associations differ across clinically relevant subgroups, including individuals with restrictive diets, malabsorption, bariatric surgery history, pregnancy, chronic disease, or medication use that may alter vitamin intake or metabolism.

## Conclusions

Higher reported dietary intakes of vitamins A, B6, and K were associated with lower odds of short sleep duration among U.S. adults in this cross-sectional analysis. These findings should be interpreted as associations and do not establish causality, therapeutic benefit, or that increasing vitamin intake would improve sleep duration or reduce chronic-disease risk. Because the analysis did not incorporate the full NHANES complex survey design, including strata and primary sampling units, the precision and statistical significance of the estimates should also be interpreted cautiously. Future studies using appropriately specified complex-survey methods, prospective designs, and more comprehensive adjustment for potential confounders are needed to confirm these associations before any clinical or dietary recommendations can be made.

## Appendices

Appendix A

NHANES 2021-2023 Sleep Disorders Questionnaire

SLEEP DISORDERS - SLQ

Target Group: 16+

The next set of questions is about {your/SP’s} sleep.

SLQ.300: What time {do you/does SP} usually fall asleep on weekdays or workdays?

|__|__| : |__|__| ENTER AM OR PM

  HH        MM

INTERVIEWER INSTRUCTION: THIS IS NOT THE TIME SP GETS INTO BED. ENTER TIME AS HH:MM AM OR PM. IF RESPONDENT SAYS TWELVE “MIDNIGHT” CODE AS 12:00 AM.

REFUSED .......................................... 77777777

DON'T KNOW ..................................... 99999999

SLQ.310 What time {do you/does SP} usually wake up on weekdays or workdays?

|__|__| : |__|__| ENTER AM OR PM

  HH       MM

INTERVIEWER INSTRUCTION: THIS IS NOT THE TIME SP GETS OUT OF BED. ENTER TIME AS HH:MM AM OR PM.

REFUSED .......................................... 77777777

DON'T KNOW ..................................... 99999999

CAPI INSTRUCTION:

SOFT EDIT: LESS THAN 4 HOURS OR MORE THAN 12 HOURS OF TOTAL SLEEP. IF SLQ.300 OR 310 IS DK OR RF, DO NOT APPLY SOFT EDIT.

ERROR MESSAGE: PLEASE VERIFY SLEEP TIMES OF LESS THAN 4 HOURS OR MORE THAN 12 HOURS.

SLQ.320 What time {do you/does SP} usually fall asleep on weekends or non-workdays?

|__|__| : |__|__| ENTER AM OR PM

  HH       MM

INTERVIEWER INSTRUCTION: THIS IS NOT THE TIME SP GETS INTO BED.

INTERVIEWER INSTRUCTION: ENTER TIME AS HH:MM AM OR PM. IF RESPONDENT SAYS TWELVE “MIDNIGHT” CODE AS 12:00 AM.

INTERVIEWER INSTRUCTION: IF RESPONDENT SAYS DOES NOT WORK, ASK IF THE TIME THAT THE RESPONDENT FALLS ASLEEP IS DIFFERENT ON WEEKENDS. IF NOT, ENTER SAME TIME AS SLQ.300.

REFUSED .......................................... 77777777

DON'T KNOW ..................................... 99999999

SLQ.330 What time {do you/does SP} usually wake up on weekends or non-workdays?

|__|__| : |__|__| ENTER AM OR PM

  HH       MM

INTERVIEWER INSTRUCTION: THIS IS NOT THE TIME SP GETS OUT OF BED.

INTERVIEWER INSTRUCTION: ENTER TIME AS HH:MM AM OR PM.

REFUSED .......................................... 77777777

DON'T KNOW ..................................... 99999999

INTERVIEWER INSTRUCTION: IF RESPONDENT SAYS DOES NOT WORK, ASK IF THE TIME THAT THE RESPONDENT WAKES UP IS DIFFERENT ON WEEKENDS. IF NOT, ENTER SAME TIME AS SLQ.310.

CAPI INSTRUCTION:

SOFT EDIT: LESS THAN 4 HOURS OR MORE THAN 12 HOURS OF TOTAL SLEEP. IF SLQ.320 OR 330 IS DK OR RF, DO NOT APPLY SOFT EDIT.

ERROR MESSAGE: PLEASE VERIFY SLEEP TIMES OF LESS THAN 4 HOURS OR MORE THAN 12 HOURS.

Appendix B

NHANES 2021-2023 Dietary Recall Questionnaire

Dietary Interview Component

Includes 24-Hour Dietary Recall Interview and Post-Recall Questionnaires

Public Health Objectives: Dietary factors are associated with 5 of the 10 leading causes of death in the U.S. population. NHANES is the cornerstone of the National Nutrition Monitoring and Related Research Program (NNMRRP). Policy makers and researchers require NHANES dietary data to assess the quality and adequacy of the U.S. diet in relation to health parameters, to evaluate the impact of program changes including welfare reform, legislation, food fortification policy, and child nutrition programs, and to identify target groups for public health education and awareness programs. Dietary practices and behaviors are used to evaluate the adoption of recommendations by the Dietary Guidelines for Americans.

The objective of the component is to estimate total intake of food energy (calories), nutrients, and non-nutrient food components from foods and beverages that were consumed during the 24-hour period prior to the interview (midnight to midnight). Following the 24-hour dietary recall, a short questionnaire is administered to ascertain whether the person’s intake on the previous day was usual or unusual, the source of tap water consumed, use of salt, special diet use, and frequency of fish and shellfish consumption during the past 30 days. In 2021-2022, the reference to the fish and shellfish hand cards was slightly reworded. These questions are included in Attachment A.

Staff: Dietary interviewer.

Protocol

Methods: All NHANES examinees are eligible for the dietary interview component. In 2021-2022, the mode of the first dietary interview changed from an in-person interview to a telephone interview to minimize face to face interaction during the COVID-19 pandemic. USDA’s Automated Multiple Pass Method is used to collect two 24-hour dietary recalls. The dietary interviews are conducted in English and/or Spanish. Data files are transmitted electronically to a coding center located offsite. For participants who completed the day 1 interview, a second dietary interview is scheduled 3-10 days later. During the mobile examination, participants schedule an appointment for the first dietary interview and receive a set of measuring guides (including a USDA food model booklet, a ruler, a set of household spoons, and a set of measuring cups and measuring spoons), an appointment reminder with the date and time of the scheduled interview, and a phone contact number. The two dietary interviews are administered via telephone. The interviewers will perform data retrieval by telephone when the information provided by the participant or a proxy is incomplete; the interviewers will obtain permission from the participant or proxy to conduct data retrieval.All dietary interviews are eligible to be digitally audio recorded. The digital recordings are reviewed to monitor the quality of the interview and provide written feedback to the interviewer (approximately 5% of each interviewer’s work will be reviewed). Verbal permission to record the interview will also be recorded. Parental consent for participants aged 12-17 will be obtained. If the participant refuses the recording, the interviewer will stop the recording. The audio recordings will be kept for three years and then destroyed.  

Time Allotment: Depending on the types and number of foods reported in the dietary recall, the length of the interview ranges from 30-40 minutes.

Health Measures: Not applicable

Eligibility:  All survey participants are eligible for the dietary interview component. Proxy

Exclusion Criteria: Circumstances that would lead to exclusion would be in instances when communication or cognitive difficulties make it impossible for the participant to provide the necessary information, and a proxy is not available to complete the interview, or the participant/proxy does not speak English or Spanish.

Justification for using vulnerable populations: Minors are included in this component because they are an important target population group. Dietary data are linked to other household interview and health component data and are used to track changes that occur in food and nutrient intakes over time. There is no reason to exclude mentally impaired or handicapped individuals because there is no contraindication.

Risks: There is no more than minimal risk associated with this component.

Report of Findings: No findings are reported to respondents.

Attachment A

POST-RECALL QUESTIONNAIRE - DRQ

Target Group: SPs Birth + (Questions grouped by age categories)

NHANES III

REC.155 Was the amount of food that {you/NAME} ate yesterday much more than usual, usual, or much less than usual?

MUCH MORE THAN USUAL ....................... 1

USUAL ......................................................... 2

MUCH LESS THAN USUAL ........................ 3

REFUSED .................................................... 7

DON’T KNOW .............................................. 9

CSFII

REC.265 When you drink tap water, what is the main source of the tap water? Is the city water supply (community water supply); a well or rain cistern; a spring; or something else?

COMMUNITY WATER .......................... 1

A WELL OR RAIN CISTERN ................ 2

A SPRING ............................................. 3

NEVER DRINK TAP WATER ............... 4

REFUSED ............................................. 7

DON’T KNOW ....................................... 9

OTHER (SPECIFY) ............................ 91

[RECORD Drinking fountain AS COMMUNITY WATER SUPPLY.]

NHANES III

REC.325 Now I'll be asking some questions about {your/NAME's} use of table salt.

What type of salt {do you/does NAME} usually add to {your/his/her} food at the table? Would you say it is ordinary salt, sea salt, lite salt, or a salt substitute?

ORDINARY, SEA, SEASONED, OR OTHER FLAVORED SALT

[includes regular iodized salt,

sea salt and seasoning salts

made with regular salt] ..................................................... 1

LITE SALT ....................................................................... 2

SALT SUBSTITUTE ........................................................ 3

NONE .............................................................................. 4 (REC.335)

REFUSED ....................................................................... 7 (REC.335)

DON'T KNOW .................................................................. 9 (REC.335)

NHANES III

REC.330 How often {do you/does NAME} add this salt to {your/his/her} food at the table? Is it rarely, occasionally, or very often?

RARELY, ....................................................................... 1

OCCASIONALLY ........................................................... 2

VERY OFTEN ................................................................ 3

REFUSED ..................................................................... 7

DON'T KNOW ................................................................ 9CSFII

REC.335 How often is ordinary salt or sea salt added in cooking or preparing foods in your household? Is it never, rarely,

occasionally, or very often?

NEVER .......................................................................... 1

RARELY ........................................................................ 2

OCCASIONALLY ........................................................... 3

VERY OFTEN ................................................................ 4

REFUSED ..................................................................... 7

DON'T KNOW ................................................................ 9

[THIS QUESTION APPLIES ONLY TO USE OF ORDINARY SALT, SEA SALT OR SEASONED SALT AND

NOT TO LITE SALT OR SALT SUBSTITUTES.]

CSFII

REC.336 This next question is about {your/NAME’s} use of salt at the table yesterday. Did {you/SP} add any salt to {your/her/his} food at the table yesterday? Salt includes ordinary salt, sea salt, lite salt, or a salt substitute.

YES .............................................................. 1

NO ................................................................ 2 (REC.340)

REFUSED .................................................... 7 (REC.340)

DON’T KNOW .............................................. 9 (REC.340)

CSFII

REC.337 What type of salt was it? (Was it ordinary salt, sea salt, lite salt, or a salt substitute?)

ORDINARY, SEA, SEASONED, OR OTHER FLAVORED SALT

[includes regular iodized salt,

sea salt and seasoning salts

made with regular salt] ................................................. 1

LITE SALT ................................................................... 2

SALT SUBSTITUTE ..................................................... 3

REFUSED .................................................................... 7

DON'T KNOW .............................................................. 9

CSFII

REC.340 {Are you/Is NAME} currently on any kind of diet, either to lose weight or for some other health-related reason?

YES................................................ 1

NO ................................................. 2 (Box 1)

REFUSED ...................................... 7 (Box 1)

DON’T KNOW ................................ 9 (Box 1)

CSFII

REC.345 What kind of diet {are you/is NAME} on?

[READ AS NEEDED: Is it a weight loss or low calorie diet; low fat or cholesterol diet; low salt or sodium diet; diabetic diet; or another type of diet?]

WEIGHT LOSS OR LOW CALORIE DIET .................... 1

LOW FAT OR CHOLESTEROL DIET ............................ 2

LOW SALT OR SODIUM DIET ...................................... 3

SUGAR FREE OR LOW SUGAR DIET ......................... 4

LOW FIBER DIET .......................................................... 5

HIGH FIBER DIET ......................................................... 6

DIABETIC DIET ............................................................. 7

LOW CARBOHYDRATE DIET ...................................... 8

HIGH PROTEIN DIET .................................................... 9

WEIGHT GAIN DIET ................................................... 10

GLUTEN-FREE OR CELIAC DIET .............................. 11

RENAL OR KIDNEY DIET ........................................... 12

OTHER ........................................................................ 91

(SPECIFY) ___________

REFUSED ................................................................... 77

DON’T KNOW ............................................................. 99BOX 1

IF SP < 1 YEAR OLD, GO TO THE END OF THE SECTION.

OTHERWISE, CONTINUE.

NHANES 1999

DRQ.361 Please look at the list of fish on the lavender hand card. During the past 30 days, did you eat any types of fish listed on this card? Include any foods that had fish in them such as sandwiches, soups, or salads.

YES................................................ 1

NO ................................................. 2 (DRQ.380)

REFUSED ...................................... 7 (DRQ.380)

DON’T KNOW ................................ 9 (DRQ.380)

NHANES 1999

DRQ.370 During the past 30 days, which types of fish did you eat and how many times did you eat them?

Type listed: breaded fish products, tuna (canned or fresh), bass, catfish, cod, flatfish, haddock, mackerel, perch, pike, pollock, porgy, salmon, sardines, sea bass, shark, swordfish, trout, walleye, other type of fish and unknown type of fish.

Interviewer instruction: Check each type of fish the SP reports eating, and then ask and record the number of times each type was eaten.

NHANES 1999

DRQ.380 Please turn the hand card over and look at this list of shellfish. During the past 30 days, did you eat any types of shellfish listed on this card? Include any foods that had shellfish in them such as sandwiches, soups, or salads.

YES................................................ 1

NO ................................................. 2 (END OF SECTION)

REFUSED ...................................... 7 (END OF SECTION)

DON’T KNOW ................................ 9 (END OF SECTION)

NHANES 1999

DRQ.390 During the past 30 days, which types of shellfish did you eat and how many times did you eat them?

Type listed: clams, crab, crayfish (crawfish), lobster, mussels, oysters, scallops, shrimp, other shellfish (for example, octopus, squid) and unknown type of shellfish.

Interviewer instruction: Check each type of shellfish the SP reports eating, and then ask and record the number of times each type was eaten.
